# The gut-liver-kidney-brain axis in Wilson disease: copper speciation-flux and barrier-mediated organ crosstalk

**DOI:** 10.3389/fimmu.2026.1840716

**Published:** 2026-06-17

**Authors:** Nannan Qian, Sihuan Zhu, Yuqi Song, Yulong Yang, Han Wang, Hui Han, Guocun Xu, Wenjie Hao, Hailin Jiang, Yue Yang, Hu Xi, Yufeng Ding, Wei He, Taohua Wei, Wenming Yang, Ting Cheng

**Affiliations:** 1 Department of Neurology, The First Affiliated Hospital of Anhui University of ChineseMedicine, Hefei, Anhui, China; 2 Graduate School, Anhui University of Chinese Medicine, Hefei, Anhui, China; 3 Key Laboratory of Xin’An Medicine, Ministry of Education, Hefei, Anhui, China; 4 Center for Xin’an Medicine and Modernization of Traditional Chinese Medicine,Institute of Health and Medicine, Hefei Comprehensive National Science Center,Hefei, Anhui, China

**Keywords:** ATP7B, bile acid metabolism, blood–brain barrier, copper flux, intestinal barrier integrity, non-ceruloplasmin-bound copper, relative exchangeable copper, renal tubular dysfunction

## Abstract

Wilson disease (WD) has long been framed as a hepatocentric disorder of copper accumulation. That view is now giving way to a broader model centered on the gut–liver–kidney–brain axis. In WD, copper is not simply stored in tissues as an inert burden. It circulates in dynamic, bioactive pools—particularly relative exchangeable copper (REC)—that disrupt barrier structures, including the intestinal epithelium and blood–brain barrier, and spread toxicity through measurable biochemical mediators. Major pathogenic processes include copper-induced suppression of autophagy, disruption of FXR-regulated bile acid signaling, and direct injury to the intestinal barrier. Gut dysbiosis, supported by fecal microbiota transplantation (FMT) studies in ATP7B-deficient mice, further amplifies hepatic inflammation and favors copper retention. Renal tubular dysfunction and neurotoxicity appear to reflect copper species-dependent passage across biological barriers together with secondary metabolic disturbances, including the recently described pathway of cuproptosis. In the clinic, this shift has been accompanied by greater use of copper-species biomarkers such as ceruloplasmin oxidase activity and REC, along with advanced imaging approaches such as ^64^Cu-PET/CT. Treatment is also moving beyond conventional chelation alone, with increasing attention to biliary copper excretion, epithelial barrier repair, and microbiome-directed interventions. Viewed in this way, the axis model helps explain the marked phenotypic heterogeneity of WD and offers a mechanistic basis for more precise interventions aimed at breaking pathogenic feedback loops across organs.

## 1 Introduction

Wilson disease (WD) remains the prototypical inherited disorder of copper metabolism, yet the familiar “copper-only” account—one that treats copper burden as a single measure of disease severity—does not explain one of the most important clinical features of WD: marked phenotypic heterogeneity. Some patients present with fulminant or acute-on-chronic liver failure and rapidly progressive systemic decline. Others develop prominent neuropsychiatric manifestations despite relatively modest hepatic symptoms, and still others show subclinical but measurable neurodegeneration in what appears clinically to be a hepatic phenotype. These divergent trajectories argue against a model in which hepatic copper accumulation leads to uniform downstream consequences. They suggest instead that phenotype depends on where bioavailable copper distributes, how individual tissues buffer copper stress, and which inter-organ amplification loops come to dominate (1–5).

Clinical observations across the literature further show that WD is not simply a liver disease with “extrahepatic complications.” It is a multi-organ disorder in which the sequence of organ involvement may depart substantially from classical teaching. Psychiatric symptoms may precede overt neurologic signs and lead to misdiagnosis, indicating that the brain phenotype can arise early and as a primary biological manifestation rather than as a secondary psychosocial response (6, 7). Neuroimaging tells a similar story. Findings range from classic diagnostic patterns to subtle abnormalities visible only on advanced sequences, suggesting that central nervous system (CNS) injury may already be present before conventional structural changes appear on routine MRI (8, 9). Systemic spillover is also evident beyond the nervous system. In drug-naïve patients, skeletal muscle imaging may show changes consistent with primary copper toxicity rather than treatment-related injury, supporting the view that extrahepatic copper exposure is clinically relevant and not confined to late hepatic failure (10).

Kidney involvement makes the same point from another angle. In pediatric acute-on-chronic liver failure cohorts that include WD, acute kidney injury is not rare and is strongly associated with worse outcomes, indicating that renal vulnerability and hepatorenal coupling can materially influence survival in severe hepatic presentations (1). The growing use of prognostic tools, including machine-learning models trained on large WD cohorts to identify early warning features of acute-on-chronic liver failure, also speaks to the heterogeneity and abrupt phase transitions of this disease—features that a static copper-burden model does not capture well (4). Taken together, these observations call for a framework that incorporates dynamic exposure states, multi-organ feedback, and measurable intermediate biology.

The gut–liver–kidney–brain axis provides such a framework, but only if it is defined in mechanistic terms and linked to testable readouts. In this model, the initiating defect in copper handling generates a circulating state of bioavailable copper that redistributes across organs. Tissue interfaces—most clearly the gut–liver interface—then amplify metabolic injury into sustained inflammation. Several of the relevant fluxes can be measured directly: copper species and copper kinetics in blood and tissues, portal delivery of microbial products, bile-acid signaling back to the intestine, and organ-specific biomarker and imaging readouts. The emphasis on flux rather than static concentration is essential. Functional copper imaging shows rapid tracer clearance from blood and substantial extrahepatic distribution, including the kidney, directly documenting systemic spillover as a measurable phenotype (11–13). In parallel, copper-species diagnostics offer a clinically usable approximation of exposure by separating total copper from more bioavailable fractions such as NCC and exchangeable copper (REC/CuEXC-derived metrics), while functional ceruloplasmin activity reflects failure of ATP7B-dependent copper incorporation (14–17).

Viewed in these terms, WD becomes a systems disorder shaped by copper flux and inter-organ crosstalk. This framework helps explain phenotypic heterogeneity by predicting that distinct clinical presentations arise from different combinations of exposure (bioavailable copper), interface amplification (for example, gut barrier failure and innate immune activation), and organ-specific vulnerability (such as mitochondrial sensitivity or excitotoxic circuitry). It also points to a more practical translational strategy: intermediate axis states become legitimate therapeutic targets and trial endpoints, rather than total copper reduction serving as the only surrogate of disease modification (17–23).

In this review, we take copper flux and copper speciation as the central organizing concepts. We examine the evidence across the gut–liver, liver–kidney, and brain interfaces, and we place validated biomarkers and testable predictions into a single translational framework for the axis model (**Supplementary Table 1**).

## 2 Copper as the central driver

### 2.1 From “Copper Load” to “Copper Flux”: why dynamics, not snapshots, govern toxicity

WD arises from defective ATP7B-mediated hepatic copper handling, but the main pathogenic driver is not simply how much copper is present in a tissue at one moment. What matters more is where copper is moving, how quickly it shifts between compartments, and which molecular carriers are involved. Together, these features define copper flux and underlie systemic spillover. Tracer studies in ATP7B-deficient models show that normal copper trafficking is fundamentally rerouted: when hepatobiliary excretion fails, copper is retained and redistributed, increasing exposure of extrahepatic tissues with distinct handling mechanisms and injury thresholds, including the intestine and kidney (12, 24). This perspective also helps explain why apparent clinical stability may coexist with ongoing subclinical injury. Redox-active mobile copper pools may continue to cycle even when conventional static markers improve, and copper-responsive transcriptional networks may remain disturbed well before advanced fibrosis becomes histologically evident (25).

Recent work has sharpened both the measurement of copper spillover and the ways it might be therapeutically modified. “Copper overload” is increasingly understood as a dynamic process of inter-organ redistribution rather than a fixed tissue concentration. One important advance has been the direct visualization of gut-to-liver copper trafficking: 64Cu-PET/CT shows rapid intestinal uptake followed by hepatic trapping, and the hepatic signal is reduced by zinc therapy, functionally identifying the gut as a controllable entry route for systemic copper exposure (26). These imaging data fit well with an axis-based view of WD, in which disease expression reflects copper movement across biological barriers and transport systems, helping explain why organ injury patterns vary despite the same underlying genetic defect (5). The idea of spillover also becomes clinically measurable when paired with toxicity-weighted copper metrics. Recent analyses suggest that relative REC better reflects systemic copper toxicity than the older and less precise construct of “free copper,” thereby linking circulating copper fractions to downstream organ risk (5). Copper entry and intracellular trafficking are also governed by specific transporters and chaperones. Rosmarinic acid, for example, inhibits hCtr1 and reduces copper influx by altering transporter localization, extending the notion of flux control from the intestinal lumen to the cell membrane itself (27). Modifier pathways add another layer: COMMD1–ATP7B interactions suggest that variation in trafficking machinery may contribute to phenotypic heterogeneity even among patients with comparable ATP7B dysfunction (28). The spillover model is further supported by parallel stress responses in different organs. In experimental copper loading, renal oxidative stress tracks closely with hepatic injury, consistent with a systemic phase in which extrahepatic organs become active targets once local buffering capacity is exceeded (29). Human imaging and biomarker studies extend this pattern to the heart, where subclinical fibrosis and strain abnormalities correlate with non-ceruloplasmin-bound copper (NCC), again pointing to multi-organ dissemination rather than isolated hepatic storage (30). At the effector level, copper stress can engage regulated cell-death programs beyond apoptosis, including ferroptosis, as suggested by GPX4 downregulation and ACSL4/ALOX15 upregulation, offering a plausible link between spillover copper pools and organ-specific injury phenotypes (31).

One practical implication is clear: disease monitoring in WD should focus less on total copper burden within a single organ and more on the toxic circulating fraction of copper and its movement across compartments. Noninvasive approaches that capture copper trafficking, including tracer imaging and kinetic modeling, provide proof of concept that flux biomarkers may track treatment response and organ-level copper handling more directly than static biochemical tests (12, 32). At the same time, mechanistic studies increasingly depict copper as a signaling disruptor. By suppressing nuclear receptor pathways, particularly FXR, copper may alter bile acid homeostasis and detoxification programs early in the disease course. This suggests that even before overt cell death develops, copper overload may rewire systemic metabolism and gut–liver communication at the transcriptional level (25).

### 2.2 Copper speciation: separating diagnostic surrogates from pathogenic carriers

Standard diagnostic approaches in WD still rely heavily on ceruloplasmin concentration and total copper measurements. Yet these measures blur the distinction between copper quantity and copper bioavailability, and they may misclassify states in which ceruloplasmin changes for reasons unrelated to copper toxicity. Functional measurement of ceruloplasmin oxidase activity more closely reflects the biologically active ceruloplasmin pool and has been proposed as a high-discrimination tool in diagnostic algorithms. More broadly, this supports the view that copper biology should be assessed by functional status and carrier specificity, not by mass alone (15, 17).

Within the axis framework, two speciation measures are particularly relevant to pathogenesis and monitoring: non–ceruloplasmin-bound copper (NCC) and relative exchangeable copper (REC). Both are intended to approximate the mobile, redox-active copper fraction most capable of reaching vulnerable tissues and participating in protein-binding and oxidative injury pathways (14, 16). REC and related exchangeable copper metrics capture weakly bound copper species that are more easily redistributed, offering a more mechanistically grounded estimate of systemic spillover risk than ceruloplasmin concentration alone (16). Clinically, these biomarkers help distinguish controlled hepatic storage from ongoing systemic dissemination, a distinction that becomes especially important when the intestine, kidney, and brain act as secondary amplifiers of toxicity during treatment (14, 16).

This shift does not diminish the value of traditional tests; it changes how they should be read. Ceruloplasmin remains useful for initial screening, but the axis model predicts that phenotypic divergence and treatment-phase instability will track more closely with fluctuations in NCC/REC-like pools and with downstream signatures of copper-driven metabolic disruption than with ceruloplasmin alone (15–17, 33). Metabolomics findings are consistent with this view. WD has recognizable metabolic signatures, and hepatic and neurologic phenotypes may show partly distinct systemic profiles, suggesting that different copper flux trajectories leave different cross-organ biochemical fingerprints (33) (**Supplementary Table 2**).

### 2.3 Systemic spillover as a trigger for node-specific injury programs

Copper toxicity is not expressed in the same way in every tissue. Different organs appear to engage distinct organelle-level injury programs in response to copper exposure. In the liver, copper overload intersects with lipid metabolic rewiring, silencing of nuclear receptor networks, and increased susceptibility to secondary bile acid stress, creating a pre-injury state in which signaling defects and metabolic bottlenecks precede or intensify overt parenchymal damage (25, 34). In the intestine, excess copper directly injures the epithelial barrier and produces histologic and ultrastructural changes that differ from those seen in hepatocytes, indicating that the gut is not merely an absorptive conduit but an early target organ in WD (20). Copper also inhibits aquaporin-3 (AQP3)-mediated permeability at micromolar concentrations, offering a plausible mechanism by which it disrupts epithelial physiology and the mucosal microenvironment (35).

The spillover model also helps connect WD with emerging concepts of regulated cell death. Although the picture is still incomplete, available data implicate non-apoptotic programs such as ferroptosis in the copper-loaded liver, suggesting that copper exposure creates conditions in which different execution pathways may be engaged depending on cellular context rather than activating one uniform death program (36). These tissue-specific injury responses become most meaningful when placed in a networked model. Copper-induced barrier damage in the intestine increases portal delivery of microbial ligands, which then reshapes hepatic immune activation, bile acid signaling, and systemic metabolite profiles. Those changes, in turn, feed back into copper homeostasis and further amplify systemic spillover (20, 25, 37, 38).

## 3 Gut node and gut–liver interface

**Figure 1:**
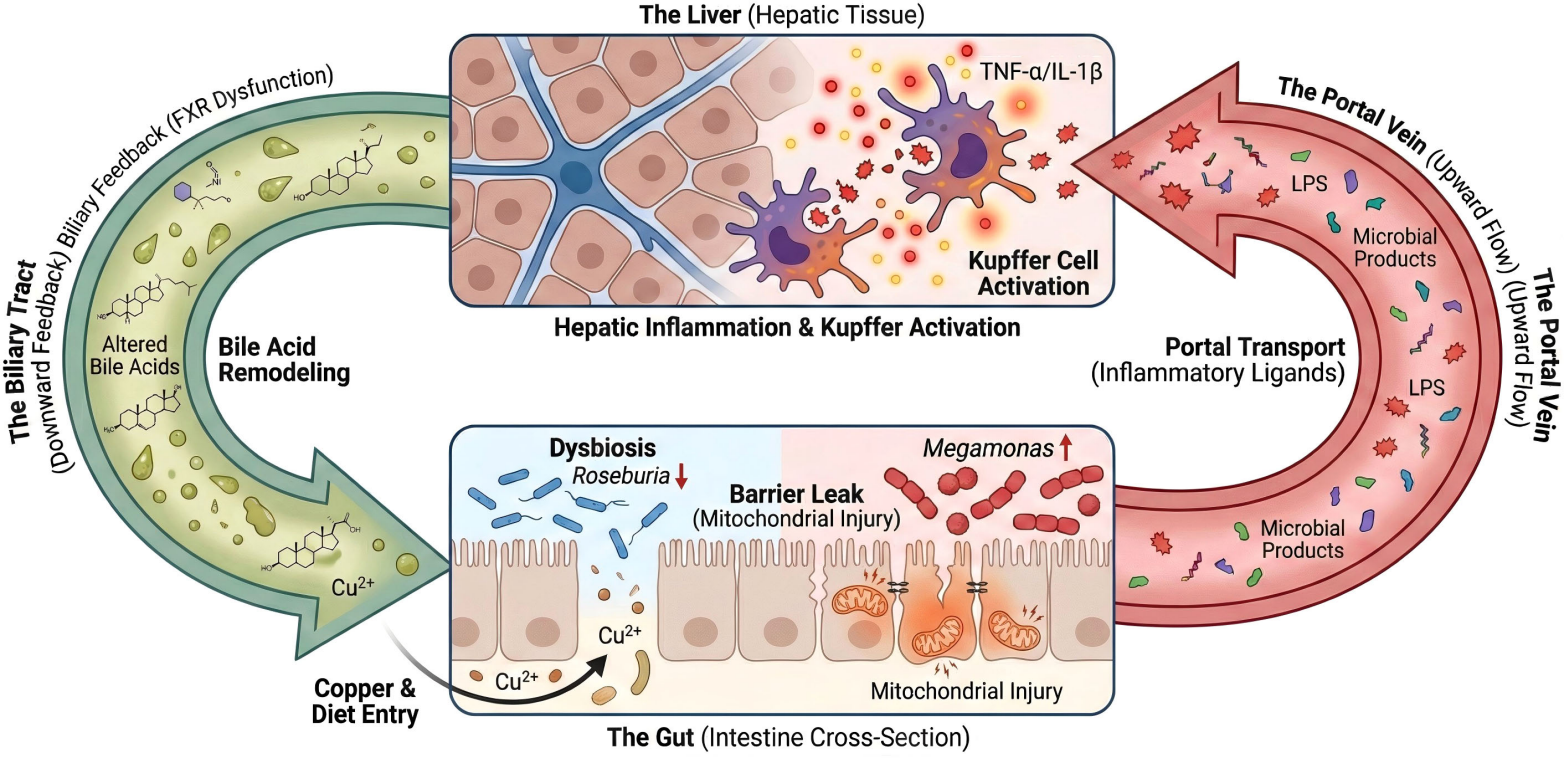
Closed-loop toxicity at the gut–liver interface in Wilson disease. Intestinal copper entry and hepatic copper accumulation promote gut dysbiosis, epithelial mitochondrial injury, and barrier leak, thereby enhancing portal delivery of lipopolysaccharide and other microbial products to the liver. These signals activate Kupffer cells and inflammatory cytokine production, while copper-induced disruption of farnesoid X receptor signaling remodels bile acids and feeds back to the intestine. The resulting gut–liver crosstalk sustains hepatic inflammation and reinforces copper-related tissue injury. AQP3, aquaporin-3; FXR, farnesoid X receptor; LPS, lipopolysaccharide; TNF-α, tumor necrosis factor-α; IL-1β, interleukin-1β.

Among all interfaces in the axis model, the gut node currently has the densest supporting evidence. It brings together three linked processes: copper-driven ecological pressure on the microbiome, direct copper injury to the epithelial barrier, and the portal circulation, which efficiently transfers mucosal disturbances to the liver. The result is a self-reinforcing loop. Copper spillover perturbs the gut ecosystem and weakens barrier integrity, increasing portal delivery of microbial products. The liver responds through innate immune sensing and altered bile acid signaling. Changes in bile composition and the luminal environment then further reshape the microbiota and worsen intestinal permeability. **Figure 1** captures this loop most clearly at the mechanistic level.

### 3.1 Dysbiosis as an axis amplifier: consistent taxonomic shifts and coupled metabolite loss

Human multi-omics studies provide direct evidence that WD is associated with a recurring pattern of dysbiosis linked to defined metabolic changes. Patients with WD show reduced microbial diversity, enrichment of taxa such as Selenomonadaceae and Megamonas, and depletion of butyrate-producing organisms including Roseburia inulinivorans, a profile consistent with a pro-inflammatory and metabolically depleted gut environment (18). These microbial shifts occur alongside loss of potentially protective fecal metabolites. Leucylproline and 5-phenylvaleric acid are reduced, whereas D-gluconic acid, a compound implicated in metal chelation, correlates with specific bacterial taxa such as Alistipes indistinctus. This pattern argues for an organized microbiota–metabolite network rather than nonspecific dysbiosis (18).

WD also stands out because evidence for microbiota involvement now extends beyond association. Integrated human and mouse studies show that Lactobacillus is consistently depleted in both WD patients and ATP7B-deficient mice, inversely correlates with the severity of liver injury, and can be restored by FMT from healthy donors. That intervention lowers hepatic copper content and improves liver injury (19). Conversely, transferring WD-associated microbiota to germ-free or antibiotic-treated recipient animals worsens hepatic injury, establishing directionality and identifying the gut microbiome as an active driver of phenotype rather than a passive bystander (19). Within the axis model, this is a particularly important observation because it suggests that correcting dysbiosis may influence not only downstream inflammation but also upstream copper homeostasis itself (19).

More recent microbiome studies strengthen this interpretation by showing that gut dysbiosis in WD is a copper-linked ecological pattern with translational relevance. In copper-loaded mice, Corynebacterium is reproducibly enriched while Lactobacillus is depleted, and treatment restores Ligilactobacillus in parallel with improvement in liver injury markers, tying a defined microbial pattern to host pathology rather than to background community variation (39). In axis terms, these data support the idea that copper spillover changes luminal and mucosal selective pressures, which then alters microbial composition and metabolic output. Rebalancing that ecology is accompanied by hepatic improvement. Dysbiosis therefore becomes both a mechanistic node and a measurable biomarker layer at the gut–liver interface (5). The same work also suggests that copper toxicity alone may not fully account for inflammatory escalation. Instead, microbial and environmental cofactors conditioned by copper exposure may determine whether early metabolic remodeling progresses to overt inflammatory injury, consistent with models in which additional “second hits” shape the inflammatory expression of copper-driven steatosis (40).

### 3.2 Barrier injury: tight junction failure and enterocyte mitochondrial vulnerability as copper-specific pathology

The clearest mechanistic evidence that copper spillover initiates the gut–liver loop comes from direct demonstrations of epithelial barrier dysfunction in both human WD samples and animal models. Duodenal biopsies from WD patients show villus blunting and lymphocytic infiltration, while rodent models display downregulation of tight-junction and adhesion proteins, including claudins and cadherins, providing a structural basis for increased intestinal permeability (20). Ultrastructural studies add a distinctive organelle-level picture: enterocytes in WD show mitochondrial swelling and loss of cristae, a pattern different from the electron-dense mitochondrial aggregates observed in hepatocytes, suggesting node-specific responses to copper exposure (20).

One of the most useful translational observations is that this injury state appears reversible. High-affinity copper chelation with methanobactin restores barrier integrity and normalizes enterocyte mitochondrial morphology, indicating that copper is not merely associated with intestinal hyperpermeability but is a direct and modifiable cause of epithelial injury (20). Copper also inhibits AQP3 at micromolar concentrations, providing a plausible molecular route by which copper disrupts epithelial function and adds osmotic and metabolic stress to the mucosal microenvironment (35). Complementing these findings, ATP7B expression and copper-dependent trafficking have been demonstrated in the stomach and small intestine, with strong localization in the duodenum and jejunum. The intestinal epithelium therefore has its own copper-handling machinery, and in WD this system may be overwhelmed, converting the gut from a passive absorption site into a direct target of copper toxicity (41).

### 3.3 From “gut leak” to hepatic inflammation: portal pattern delivery and Kupffer cell activation under copper-shaped immunity

Once the barrier fails, the portal vein becomes an efficient route for microbial-associated molecular patterns (MAMPs), especially LPS/endotoxin, to reach the liver and activate innate immune receptors on resident macrophages. Direct measurement of portal endotoxin flux in WD remains limited, but human and animal data converge on persistent hepatic innate immune activation that fits the proposed sequence of barrier leak, portal delivery, and Kupffer cell activation. In treated asymptomatic WD patients, soluble CD163 (sCD163), a marker of Kupffer cell activation, remains elevated and inversely correlates with metabolic liver function measured by galactose elimination capacity, yet does not correlate with FibroScan-defined fibrosis. This separation suggests an immune–metabolic injury axis that can progress independently of structural scarring (38).

Endotoxemia models further show that copper dyshomeostasis reshapes hepatic immune responses during LPS challenge. In LPS-induced endotoxemia, ATP7B-deficient mice have greater neutrophil recruitment to hepatic sinusoids but impaired neutrophil extracellular trap (NET) formation, a paradox that points to copper-driven dysfunction of antimicrobial effector programs. Rising gut-derived ligand delivery may therefore intensify inflammatory injury while at the same time weakening pathogen containment (42). Cell-specific knockout experiments support the idea that extrahepatocytic signals are needed for full inflammatory progression. Hepatocyte-specific loss of ATP7B causes marked copper accumulation but only mild inflammation and delayed disease onset, implying that intracellular copper retention alone is not enough to trigger robust inflammatory cascades. This is consistent with the axis model, which predicts that gut-derived mediators and non-parenchymal immune sensing are required to amplify injury (43).

### 3.4 Liver-to-gut feedback: bile acids, FXR dysfunction, and luminal remodeling that close the loop

A defining feature of the axis model is bidirectionality. The liver does not simply receive gut-derived signals; it also shapes the gut environment through bile acid secretion and immune signaling, and copper overload disrupts that output in ways that favor dysbiosis and barrier failure. In ATP7B-deficient liver, copper accumulation reduces FXR chromatin binding and contracts the FXR cistrome, leading to downregulation of canonical targets such as BSEP and SHP. The result is bile acid retention and altered bile acid composition in both serum and liver tissue. Comparable abnormalities have been reported in the serum of WD patients and correlate with the severity of liver disease, making bile acid profiling a functional readout of impaired liver-to-gut signaling (37). Proteomic systems studies support the same mechanism. They show early suppression of nuclear receptor networks, including FXR, before advanced histopathology develops, suggesting that copper acts as a transcriptional disruptor that alters bile acid transport and detoxification and thereby changes luminal bile pressure and microbial ecology (25). Clinically, serum total bile acids independently predict severe liver disease, reinforcing their value as an axis-relevant marker rather than a nonspecific consequence of cirrhosis (44).

Bile acids may also serve as a synergistic second hit in the copper-loaded liver, tightening the feedback loop still further. In ATP7B-deficient hepatocytes, cholic acid feeding aggravates oxidative injury and apoptosis, indicating that copper-laden cells are unusually sensitive to bile acid stress (34). Taken together, these findings support a closed-loop view of the gut–liver interface: copper spillover promotes dysbiosis and barrier leak, portal translocation of microbial products drives hepatic innate immune activation, and disturbed bile acid signaling feeds back to remodel the gut ecosystem. This self-sustaining cycle is the core mechanism summarized in **Figure 1** (42–44).

## 4 Liver–kidney interface

### 4.1 Clinical spectrum: from proximal tubulopathy to distal acidification failure

Renal involvement in WD has traditionally been viewed as a secondary consequence of systemic copper overload. Increasingly, however, the kidney appears to be a true axis node with its own vulnerability and with the capacity to reinforce systemic injury once damaged. Clinically, renal manifestations range from proximal tubular dysfunction, classically presenting as Fanconi syndrome with low-molecular-weight proteinuria, aminoaciduria, phosphaturia, glycosuria, and electrolyte wasting, to distal nephron dysfunction characterized by impaired urinary acidification, distal renal tubular acidosis (dRTA), nephrocalcinosis, and metabolic bone disease. Importantly, dRTA may be the first or dominant presentation, challenging the traditional idea that Fanconi syndrome comes first and broadening the clinical entry point for diagnosing WD (45–47). Pediatric observational studies also show clinically meaningful rates of hypercalciuria and microalbuminuria, findings consistent with early tubular and glomerular stress that may precede overt hepatic or neurologic disease and may continue to amplify systemic injury even after hepatic indices improve (46). Nephrolithiasis should also be incorporated into the axis model. Stones occur in WD cohorts and may newly develop or persist despite treatment, even without marked hypercalciuria, suggesting longstanding tubular injury or ongoing subtle dysregulation of copper flux and tubular handling (48).

At the same time, primary WD-related renal injury must be distinguished from treatment-related nephrotoxicity, because medications can produce glomerular and vascular lesions that mimic or worsen native tubulopathy. Case reports and mechanistic studies have linked D-penicillamine to membranous nephropathy and other proteinuric syndromes, and additional reports describe ANCA-associated vasculitis during therapy (49–52). Renal dysfunction in WD therefore needs to be interpreted within an integrated axis framework that considers both disease-driven and treatment-driven mechanisms, rather than assigning every renal abnormality to a single cause (53).

### 4.2 Mechanistic susceptibility: copper handling, tubular energetics, and a plausible “cuproptosis” bridge

The renal phenotype in WD is broader than classical tubulopathy and may precede the usual hepatic or neurologic presentation. Early-onset cases show that copper-associated proximal tubular dysfunction can manifest as hypercalciuria and nephrocalcinosis before liver failure, making otherwise unexplained stones or nephrocalcinosis potential clues to WD (50). Distal tubular dysfunction can add further morbidity. Gross hematuria may occur downstream of dRTA-related alkaline urine and calcium precipitation, increasing stone burden and urothelial injury in susceptible patients (54). These renal manifestations also complicate diagnosis. Significant proteinuria, for example, may artifactually lower serum ceruloplasmin and increase urinary copper excretion, making genetic testing or quantitative hepatic copper measurement necessary to avoid misclassification (55). Experimental copper loading further shows that renal oxidative stress parallels hepatic antioxidant depletion, supporting the view that the kidney is an active target organ in systemic copper toxicity rather than a passive excretory conduit (29). Clinical experience with extracorporeal albumin dialysis (MARS) in fulminant WD is consistent with this interpretation: reducing circulating copper improves both encephalopathy and renal function, highlighting the tight coupling between copper burden and multi-organ dysfunction in acute disease (56).

Renal remodeling also deserves attention. In TX mice, renal interstitial fibrosis is associated with activation of the leptin–JAK2–STAT3 pathway, and pharmacologic inhibition of this pathway attenuates fibrotic injury (57). This provides a defined molecular route by which copper stress may drive chronic kidney remodeling and supports earlier renal-protective strategies within multi-organ WD management. More broadly, these findings suggest that variable tubular phenotypes, diagnostic interference, and progressive fibrotic signaling may all contribute to systemic disease progression, in line with the wider view that WD affects matrix biology and developmental programs beyond the liver (58, 59).

Mechanistically, the kidney is not simply filtering copper. Renal tubules express active copper transport and buffering systems that may become maladaptive during systemic spillover. Physiologic studies show co-expression of ATP7A and ATP7B in renal tubules, and in ATP7B deficiency, altered ATP7A trafficking appears to modify basolateral copper reabsorption. This offers a plausible explanation for interpatient variability in tubular phenotypes and for the persistence of hypercalciuria as a stable renal signature even when hepatic disease dominates the clinical picture (60). Tissue-mapping studies showing region-specific copper-handling machinery throughout the gastrointestinal tract and other organs reinforce the idea of a distributed flux network rather than a strictly liver-centered model (41). Copper-mediated inhibition of AQP3 in the collecting duct provides another plausible link between copper exposure and impaired water handling, potentially worsening acidification defects in susceptible patients (35).

Whether cuproptosis contributes directly to renal tubular injury in WD remains an open question. Copper-triggered, FDX1-dependent lipoylation stress has been described in tubular epithelial injury outside WD (61), but direct evidence in ATP7B deficiency is still lacking. For that reason, cuproptosis should not yet be treated as an established renal mechanism in WD. A more definitive test would likely require WD-specific proximal tubule organoid models. Such systems could preserve the apical–basolateral polarity needed for copper transporter trafficking and would allow direct assessment of whether Fanconi-like tubulopathy in WD reflects cuproptosis or some other form of metabolic collapse (60, 61).

### 4.3 Feedback: how kidney dysfunction can sustain systemic injury and reshape other nodes

Once tubular dysfunction develops, it may feed back into systemic homeostasis through several axis-relevant routes even before chronic kidney disease becomes advanced. Acid–base and electrolyte disturbances can alter intestinal luminal chemistry and microbial metabolism, thereby engaging the gut node. Reduced excretory capacity may increase circulating uremic solutes and inflammatory mediators, and recurrent stone risk or persistent subclinical tubular injury may create a chronic source of metabolic instability that slows hepatic recovery (46, 48, 62). In this sense, the kidney is better viewed not as a passive endpoint but as an active amplifier. Renal injury may prolong systemic exposure to toxic metabolites and immune stimuli, lowering the threshold for hepatic inflammation or neurologic decompensation during periods of fluctuating copper mobility, including treatment initiation or escalation (62, 63). This has practical therapeutic implications: copper-sequestering strategies that reduce bioavailable copper without promoting redistribution may help relieve both brain stress and renal tubular stress. Clinical development of tetrathiomolybdate-class agents offers a useful basis for testing this possibility in an axis-informed framework (53, 64).

## 5 Brain interfaces

### 5.1 Liver–brain interface: direct evidence for copper flux to the CNS, barrier injury, and imaging-defined neurotoxicity

The liver–brain interface in WD is not simply a conceptual extension of liver disease; it is supported by defined transport pathways and measurable neuropathological consequences. Barrier transport studies show that systemic copper can enter the CNS through both the blood–cerebrospinal fluid (CSF) barrier at the choroid plexus and the blood–brain barrier (BBB). Copper uptake is rapid at the blood–CSF interface, whereas transfer across the BBB is slower but still substantial because of the barrier’s large surface area. These routes provide a concrete anatomical basis for rising bioavailable copper to reach neural tissue during systemic spillover (65, 66). The neurovascular barrier is also an active target of injury. *In vitro* BBB co-culture systems show that circulating copper–albumin complexes disrupt tight-junction architecture, particularly ZO-1 and occludin, whereas bis-choline tetrathiomolybdate sequesters copper into inert complexes and preserves barrier integrity (34). This offers one explanation for why copper-sequestering agents may carry a lower risk of redistribution-related neurotoxicity than classical chelators, which can transiently raise free copper. Supporting this flux-based interpretation, toxic milk mouse studies show that penicillamine initiation rapidly increases serum free copper, raises brain copper content, alters transporter expression, and intensifies oxidative stress markers, providing a causal basis for paradoxical worsening at treatment onset (34, 63).

Clinical biomarker studies further support the relevance of BBB dysfunction and glial-interface injury in WD (67, 68). Serum markers reflecting endothelial activation, extracellular matrix remodeling, astroglial reactivity, and BBB compromise — including ICAM-1, P-selectin, MMP-9, GFAP, and S100B — have been profiled in untreated and treated WD cohorts and shown to correlate with neurological impairment and treatment response (67, 69). Notably, GFAP elevation appears largely restricted to untreated patients with the neurological phenotype and decreases during anti-copper therapy, consistent with reversible astroglial activation (67). Although these markers have not yet been validated as routine clinical tools (70), they provide an important translational bridge between experimentally demonstrated BBB injury, dysregulated copper speciation (71), and clinical neurological progression. Future longitudinal studies should therefore integrate BBB-related biomarkers with NfL (72), copper-species quantification (e.g., exchangeable and relative exchangeable copper) (71), and quantitative MRI (73) to determine whether barrier dysfunction causally mediates the transition from systemic copper spillover to overt neurodegeneration.

Traditional descriptions of brain MRI in Wilson disease have included visually recognizable signs such as the “face of the giant panda”, as well as occasional white-matter abnormalities and ventricular enlargement related to regional atrophy (9). However, these findings are neither frequent nor truly pathognomonic, and should be interpreted as supportive but non-specific imaging clues, particularly in patients with neurological WD. Contemporary neuroimaging assessment should therefore move beyond rare symbolic signs and focus on reproducible lesion distribution, quantitative lesion burden, regional atrophy, diffusion, susceptibility, perfusion changes, and their correlation with neurological severity and copper-related biomarkers (74). Large cohort MRI studies in neurologic WD have refined the spatial map of lesions and support standardized pattern recognition as a practical indicator of disease burden (75). A validated semiquantitative MRI scale separates potentially reversible features of acute toxicity, such as edema and T2 hyperintensity, from more chronic features such as atrophy and hypointensity, and correlates closely with clinical severity (76). High-field quantitative susceptibility mapping (QSM) in WD mouse models further suggests that copper deposition produces a diamagnetic signature distinct from the paramagnetic effects of iron, raising the possibility of a noninvasive “virtual biopsy” for copper rather than a nonspecific readout of metal accumulation (77). Multimodal MRI, including susceptibility-weighted imaging (SWI) combined with arterial spin labeling (ASL), can detect occult metal deposition and regional hypoperfusion even when standard structural sequences appear normal, reinforcing the idea that flux-driven neurotoxicity may first appear as functional disturbance rather than gross structural injury (8).

Importantly, CNS involvement in WD should not be judged solely on the basis of overt neurological symptoms or conspicuous MRI findings (78). Blood-based markers of neuroaxonal injury add a more sensitive layer of evidence for detecting and tracking brain involvement (78, 79). In WD, serum or plasma neurofilament light chain (NfL) has been associated with neurological phenotype and clinical severity (79, 80), with MRI-defined structural damage (73, 80), and with the risk of early neurological deterioration after treatment initiation (72). These observations are particularly relevant to hepatic and clinically non-neurological forms of WD, as they indicate that subclinical neuroaxonal injury may already be ongoing before classical neurological syndromes or overt MRI changes become apparent (78, 79). Within an axis-based framework, NfL may therefore act as a downstream readout that integrates systemic copper dyshomeostasis, neurovascular vulnerability, and evolving CNS injury (78, 81), providing a quantitative bridge between peripheral pathology and central nervous system damage.

Recent neurology-focused work has also made clear that phenotypic heterogeneity and diagnostic delay are intrinsic features of WD neurobiology and may be reduced by imaging-guided, mechanism-aware diagnostic pathways. WD remains one of neurology’s great mimics. A 2025 review highlights frequent misclassification as parkinsonism, dystonia, or primary psychiatric disease, and notes that ceruloplasmin may appear misleadingly normal during systemic inflammation, delaying diagnosis in patients already developing subclinical neurotoxicity (82). Newer imaging studies also argue against a single stereotyped basal ganglia pattern. Ultra-high-field 7T SWI identifies a mixed putaminal signal—iron-like hypointensity with a hyperintense rim suggestive of an additional diamagnetic component—that may help distinguish WD from other metal-related neurodegenerative disorders (83). Connectome-gradient analyses show macroscale network compression with transcriptomic enrichment of mitochondrial and synaptic pathways in affected regions, linking molecular susceptibility to systems-level dysfunction (84). Additional trial-ready markers are emerging. A weighted diffusion-weighted imaging (DWI) scale correlates more closely with neurologic severity than conventional MRI scoring, and reversible DWI tract hyperintensities after decoppering suggest that at least some lesions reflect treatable intramyelinic edema rather than fixed necrosis (85, 86).

This heterogeneity is also evident in lesion distribution and clinical presentation. Symmetric brainstem-predominant T2/FLAIR abnormalities may occur even when the basal ganglia are relatively spared (87, 88). Some patients present with isolated cerebellar atrophy and are initially misclassified as having spinocerebellar ataxia until ATP7B mutations are identified (89). Epilepsy, often generalized tonic–clonic and associated with cortical or subcortical lesions, further shows that cortical involvement can dominate in selected subgroups, including patients with paradoxical symptom onset after treatment begins (90). At the mechanistic level, work outside WD has raised the possibility that cuproptosis-related machinery may help explain why metabolically active neurons are susceptible to copper-induced cell death (91). Therapeutically, oxidative stress appears to be a modifiable component of neurologic disease. One clinical study reported that integrative treatment lowered malondialdehyde (MDA), increased superoxide dismutase (SOD), and improved dysphagia scores, supporting redox modulation as a plausible adjunct in neurorehabilitation (92).

Recent morphometric studies also weaken the conventional separation between “hepatic” and “neurologic” WD. In patients classified clinically as having hepatic WD, brain morphometry shows occult atrophy in the thalamus, globus pallidus, hippocampus, and brainstem, indicating that structural neurodegeneration may precede overt neurologic symptoms and supporting the idea that the brain is exposed once mobile copper increases, even before a clear neurologic syndrome emerges (2). Automated volumetric MRI studies further link thalamic and cerebellar atrophy to cognitive impairment, suggesting that regional volume loss is a quantitative marker of the cognitive–motor phenotype rather than a late epiphenomenon (3, 93). Similar structure–function coupling is seen in brainstem-predominant disease, where eye movement abnormalities correlate with midbrain and pontine atrophy, making oculomotor metrics a potentially sensitive clinical readout of brainstem degeneration and copper flux-related injury (94). Together, these findings support a view of the liver–brain interface as a dynamic interaction between copper flux and barrier integrity, with imaging endpoints that could serve as primary or secondary outcomes in axis-based trials (**Supplementary Table 3**).

### 5.2 Gut–brain interface: emerging and indirect links grounded in WD dysbiosis and barrier biology

Compared with the liver–brain interface, evidence for a gut–brain pathway in WD is still indirect. Even so, the axis model gives it biological plausibility because the gut can influence systemic inflammation, metabolite availability, and barrier integrity, all of which may alter the threshold for neurotoxicity. Human and translational multi-omics studies show WD-associated dysbiosis with depletion of butyrate-producing taxa and loss of potentially protective metabolites. In parallel, animal studies indicate that restoration of Lactobacillus abundance through FMT lowers hepatic copper and improves liver injury. Together, these data support a cascade in which microbiome configuration affects copper homeostasis and systemic inflammatory tone, thereby potentially modifying brain exposure and vulnerability to copper toxicity (18, 19). Independent histopathologic studies also show villus blunting, tight-junction disruption, and enterocyte mitochondrial swelling in WD, all of which are reversible with high-affinity copper chelation, indicating that gut leak is a copper-responsive pathological state rather than a fixed comorbidity (20).

A reasonable working hypothesis, then, is that copper-driven dysbiosis and barrier failure increase portal and systemic delivery of microbial ligands and inflammatory mediators, which may prime neuroinflammatory pathways or weaken BBB resilience, lowering the threshold for symptomatic neurotoxicity even when cerebral copper deposition accumulates slowly. Persistent elevation of Kupffer cell activation markers such as sCD163 in treated asymptomatic WD, together with copper-shaped immune responses in endotoxemia models, provides biologically coherent intermediate steps between gut-derived signaling and systemic immune programming (38, 42). That said, direct evidence in WD models showing that this sequence alters BBB permeability or microglial activation is still missing. For now, the gut–brain interface should be regarded as a well-reasoned but still inferential construct. Definitive support will require longitudinal studies that directly trace the path from barrier failure to BBB compromise, and that level of evidence is not yet available in the WD literature (38, 42).

### 5.3 Kidney–brain interface: analogous to CKD models and positioned as a testable WD hypothesis

Kidney-to-brain coupling in WD is best regarded as a testable hypothesis grounded in axis logic and in what is already known from related renal disorders, rather than as an established WD-specific mechanism. The rationale has two main parts. First, renal tubulopathy in WD produces chronic acid–base disturbances, electrolyte imbalance, and stone-related inflammatory stress, each of which could affect neuronal excitability, cerebrovascular tone, or systemic inflammation and thereby alter susceptibility to copper-related neurotoxicity (45, 46, 48). Second, work in chronic kidney disease (CKD) has already shown that uremic toxins and renal-derived inflammatory mediators can drive systemic and CNS dysfunction. WD may recapitulate part of this biology even without advanced CKD if tubular injury is persistent and metabolic instability is sustained (62).

From this perspective, WD-related renal dysfunction may act as a permissive amplifier of neurologic injury by increasing circulating uremic solutes, altering gut microbial metabolism through acid–base shifts, and raising systemic inflammatory tone, together weakening BBB integrity or intensifying oxidative stress responses to copper exposure. This possibility is especially relevant during treatment phases, when redistribution-related surges in free copper may be more damaging in a system already stressed by renal-derived metabolic disturbance. It also argues for monitoring strategies that integrate renal biomarkers, copper speciation, and neuroimaging rather than treating each organ system in isolation (34, 62, 63).

## 6 Integrating the axis: from evidence matrix to testable predictions

The main value of the axis framework is not that it broadens the story of WD, but that it turns the disease into a series of testable intermediate states between copper dyshomeostasis and overt organ injury. When the evidence is organized by interface, three patterns stand out. The strongest causal chain is at the gut–liver interface, where convergent human multi-omics data and interventional animal studies support a sequence in which copper spillover drives dysbiosis and barrier dysfunction, followed by portal delivery of microbial products and inflammatory mediators that amplify hepatic injury (95–102). Liver–kidney coupling is clinically important but remains less well resolved mechanistically: renal tubular vulnerability often accompanies severe disease and likely shapes systemic toxicant handling as well as redox and inflammatory tone, yet direct interventional studies in WD models remain sparse (103–116). One axis is relatively well supported, with evidence from WD and copper-overload models linking direct copper toxicity to neural injury and neuroinflammation (117–119), alongside clinical and MRI-based studies demonstrating consistent structural, quantitative, and radiomics-defined brain abnormalities in WD cohorts (120–124). By contrast, the alternative model involving gut- and kidney-derived systemic amplification is increasingly plausible on the basis of WD metabolomic and biomarker studies, but it still rests more on inference and triangulation than on definitive causal evidence within WD cohorts (125, 126).

This structure also points to a tiered biomarker strategy in which the readouts reflect axis states rather than isolated organ damage. At the exposure level, copper-species measures—including NCC and exchangeable copper (CuEXC) metrics such as REC—together with functional ceruloplasmin oxidase activity, provide information on bioavailable copper and should therefore track systemic spillover more closely than total copper alone (12–17). At the interface level, gut microbial profiles, fecal and serum metabolite signatures, barrier markers, tight-junction integrity measures, hepatic innate immune activation indices, and bile acid profiles together quantify the strength of the gut–liver loop and its liver-to-gut feedback arm (96–98). At the organ-phenotype level, quantitative neuroimaging and noninvasive fibrosis assessment allow longitudinal follow-up of downstream consequences and offer realistic target-engagement endpoints for interventional studies (127).

## 7 Therapeutic implications: multi-node interruption of the axis

### 7.1 Restoring physiologic copper exit and reducing systemic spillover

**Figure 2:**
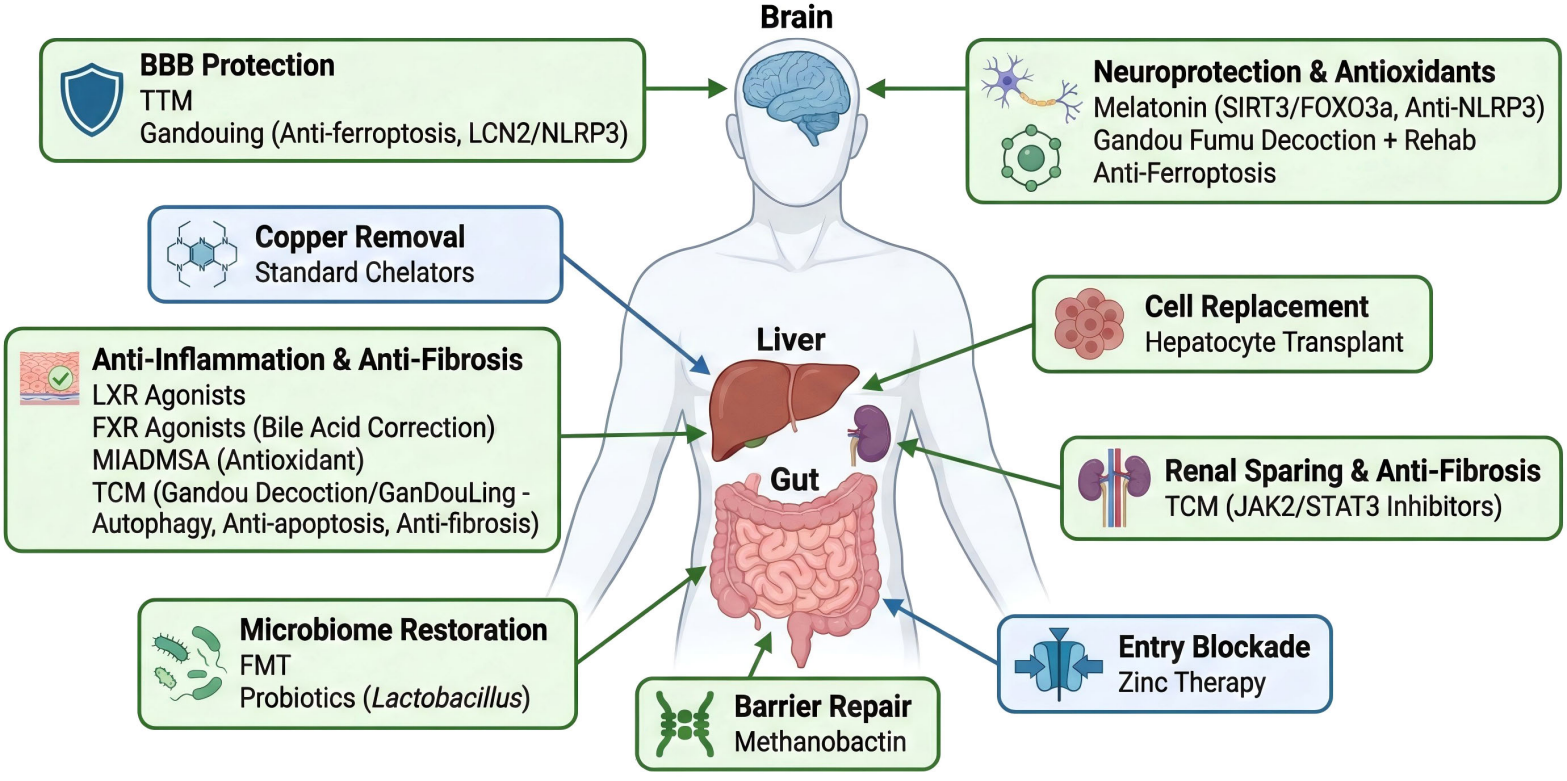
Therapeutic target map across the gut–liver–kidney–brain axis in Wilson disease. Schematic overview of representative therapeutic strategies targeting key nodes of the gut–liver–kidney–brain axis in Wilson disease. Gut-directed approaches include zinc therapy to limit copper entry, microbiome restoration by fecal microbiota transplantation or probiotic supplementation, and barrier repair represented by methanobactin. Liver-directed strategies include standard copper chelation, anti-inflammatory and anti-fibrotic interventions, modulation of farnesoid X receptor and liver X receptor signaling, traditional Chinese medicine-based formulations, and hepatocyte replacement. Kidney-directed interventions aim to reduce renal fibrotic remodeling, whereas brain-directed approaches emphasize blood–brain barrier protection, antioxidant pathways, and suppression of neuroinflammatory injury. Collectively, these strategies illustrate how therapeutic intervention can be distributed across multiple organs and pathogenic processes, rather than focusing exclusively on copper removal. BBB, blood–brain barrier; FMT, fecal microbiota transplantation; FXR, farnesoid X receptor; LXR, liver X receptor; NLRP3, NLR family pyrin domain containing 3; SIRT3, sirtuin 3; FOXO3a, forkhead box O3a; STAT3, signal transducer and activator of transcription 3; TTM, tetrathiomolybdate.

The axis model reframes copper reduction as necessary but not always sufficient. Once downstream metabolic and inflammatory programs become self-sustaining, lowering copper alone may not fully collapse the pathogenic loop (128–132). Therapies that restore physiologic copper elimination, especially through hepatobiliary export and fecal excretion, are therefore particularly attractive because they reduce systemic spillover at its source (132–134). Methanobactin currently provides the clearest experimental proof of concept in ATP7B-deficient models, rapidly mobilizing hepatic copper into bile and restoring fecal copper excretion, with multimodal imaging supporting true flux restoration rather than simple redistribution (20). This may matter beyond the liver, because reducing reliance on renal clearance could also lessen tubular stress during intensive decoppering (109, 110, 112). Bis-choline tetrathiomolybdate (TTM) may offer a complementary advantage. *In vitro*, it preserves BBB tight-junction integrity by sequestering copper–albumin complexes, providing a mechanistic basis for a lower risk of neurologic worsening than chelators that transiently increase redox-active copper (95, 123). Zinc remains a core therapy for reducing intestinal copper absorption and may work particularly well when paired with biomarker-guided strategies directed at bioavailable copper fractions (135–139) (**Figure 2**).

### 7.2 Gut node targeting: microbial remodeling and barrier repair

Gut-directed therapy in WD is no longer purely speculative. Interventional microbiome studies now provide direct causal support. In ATP7B-deficient models, FMT from healthy donors restores depleted Lactobacillus, lowers hepatic copper burden, and attenuates liver injury, whereas transfer of WD-associated microbiota aggravates hepatic pathology (19). These results indicate that the gut microbiome can influence both copper homeostasis and inflammatory signaling. Human multi-omics studies add another layer by showing that depletion of butyrate-producing taxa such as Roseburia coincides with loss of potentially protective metabolites, making microbial and metabolic restoration plausible axis-directed strategies rather than generic adjunctive measures (18).

Barrier repair is a complementary therapeutic target because axis amplification depends on leak-mediated portal transfer of microbial products. Experimental studies show that copper toxicity disrupts tight-junction architecture and enterocyte mitochondrial homeostasis, whereas interventions that reduce toxic copper flux partly restore epithelial integrity and normalize inflammatory signals (97, 98, 102). In other words, flux control and barrier stabilization converge on the same pathogenic node and may be synergistic when pursued together. Because bile acids form a key liver-to-gut feedback signal, strategies that normalize bile acid signaling and FXR-related pathways may also help stabilize gut ecology and barrier function. WD-specific interventional evidence is still less developed than in other cholestatic or metabolic liver diseases, but the mechanistic rationale is strong enough to justify including these pathways in axis-based trial design (44, 96).

### 7.3 Liver node: inflammation control and fibrosis reversal as loop dampening

At the liver node, treatment efficacy should be judged not only by reductions in hepatic copper but also by the extent to which innate immune activation and fibrogenic remodeling are dampened. These processes help sustain systemic inflammation and influence vulnerability in extrahepatic organs. Evidence implicating Kupffer cell/macrophage activation, oxidative injury, and canonical profibrotic pathways therefore identifies several mechanistic entry points for adjunctive therapy beyond standard chelation (29, 38, 140).

Importantly, the availability of noninvasive monitoring tools — including transient elastography combined with serum indices such as APRI and FIB-4 — makes longitudinal assessment of fibrosis regression at the liver node practically feasible. This matters in axis terms because fibrosis alters bile acid flow, immune tone, and portal hemodynamics, all of which feed back into gut–liver crosstalk (127).

### 7.4 Brain node: BBB protection, neuroinflammation, and metalotoxic cell-death programs

At the brain node, two therapeutic goals stand out. One is to preserve BBB and related interface integrity so that neurotoxic exposure states are less able to trigger neuroinflammation. The other is to interrupt metal-triggered cell-death programs that may continue to drive neurodegeneration even after systemic copper indices improve. The observation that copper–albumin complexes directly damage BBB tight-junction proteins, and that bis-choline tetrathiomolybdate (TTM) can neutralize this effect, provides a concrete basis for a BBB-centered neuroprotective strategy within the axis framework (34).

Beyond barrier preservation, regulated cell-death pathways, including ferroptosis-associated inflammatory cascades, are emerging as plausible downstream convergence points for copper stress, oxidative injury, and immune activation (34, 140, 141). This makes them attractive targets for mechanistic studies and for biomarker development. Experimental WD models have begun to identify pharmacologically tractable nodes at this intersection. Modulation of LCN2/NLRP3 signaling has been associated with attenuation of ferroptosis and neuroinflammation in copper-loaded models, supporting an axis-based view in which neuroprotection lies at the intersection of metal dyshomeostasis and innate immunity rather than simple metal deposition (141). Quantitative neuroimaging measures that capture reproducible anatomic and microstructural patterns in WD should therefore be incorporated as downstream organ-phenotype readouts in future trials aimed at upstream axis components, making it possible to test whether changes in flux and interface biology mediate neurologic outcomes (121).

### 7.5 Targeting oxidative stress and fibrosis: multi-component pharmacology and target engagement

A recurring criticism of multi-component therapies is that their mechanisms are too diffuse to test rigorously. This concern is being addressed by methodological work that identifies bioactive constituents and candidate pathways with greater precision. High-resolution mass spectrometry has characterized active compounds in candidate multi-component formulations, providing a molecular basis for antioxidant and anti-inflammatory effects and enabling targeted follow-up studies, including docking and pathway-enrichment analyses, rather than reliance on purely empirical claims (142). Network pharmacology studies have likewise highlighted modules such as PI3K/Akt that can be tested directly as axis-relevant links between redox regulation, cell survival, and fibrogenesis (143).

Some natural products may also act upstream by reducing copper entry rather than chelating it after intracellular accumulation. Rosmarinic acid, for example, inhibits the copper importer hCtr1 through a ternary-complex mechanism that interferes with membrane trafficking, fitting well with the concept of flux interception at the point of cellular entry (27). Other compounds, such as curcumin, show hepatoprotective effects in copper-loaded models by reducing lipid peroxidation, restoring endogenous antioxidant systems including GSH, and suppressing pro-inflammatory cytokines (142). The broader implication is that multi-component therapies should be judged less by whether they appear globally beneficial and more by which axis nodes they affect and whether they measurably reduce pathological loop gain when paired with biomarker-based readouts (104, 142).

Multi-component formulations whose chemical composition has been resolved by high-resolution mass spectrometry can be evaluated as mechanistically testable adjuncts to copper chelation rather than as nonspecific supportive therapies. Early experimental studies show lower aminotransferase levels and improved histopathologic injury after such treatment (127). Systems-level work has added more detail: RNA-seq in TX mice indicates that such formulations reshape hepatic transcriptional programs enriched for PI3K–Akt and PPAR signaling as well as ECM–receptor interaction pathways, linking these adjuncts to metabolic remodeling and matrix regulation (144). Complementary network pharmacology and *in vivo* validation studies point in the same direction, with PI3K/Akt activation and Bax/Bcl-2 modulation consistent with enhanced hepatocyte survival and reduced stellate cell-driven fibrogenesis (145).

More clinically oriented studies report significant reductions in serum fibrosis markers, including hyaluronic acid (HA), laminin (LN), procollagen type III N-terminal peptide (PCIII), and collagen type IV (IV-C), during integrative treatment, supporting a possible antifibrotic effect in human disease management (146). One study further combined FibroTouch with serum indices such as APRI and FIB-4 to create a practical noninvasive framework for longitudinal monitoring of fibrosis regression in real-world settings (127). Mechanistic work continues to expand. More recently, attention has turned to regulated lipid peroxidation-dependent cell death: copper burden is associated with a ferroptosis-like signature characterized by GPX4 downregulation and ACSL4/ALOX15 upregulation, and such formulations appear to attenuate this pattern (31). A randomized, double-blind, placebo-controlled trial has further reported that an antifibrotic multi-component intervention modified intestinal flora and improved liver fibrosis indices in WD patients (101), providing peer-reviewed RCT-level evidence that gut–liver axis modulation is a tractable therapeutic strategy in WD. Taken together, these studies support placing such well-characterized multi-component therapies within the gut–liver–kidney–brain axis as node-directed adjuncts that may influence matrix turnover, redox balance, autophagy, and ferroptosis. They also offer concrete biomarker and imaging-compatible readouts for testing target engagement alongside standard decoppering therapy (101, 146).

## 8 Knowledge gaps and future directions: moving from correlation to causal axis medicine

Current WD research still leans heavily on narrative inference. That tendency obscures mechanism beneath layers of association. The gut literature is a clear example: descriptive multi-omics datasets are now abundant, but studies that rigorously separate correlation from causation remain uncommon. The next step for the field is not more cataloging alone, but perturbation-based designs that can determine whether microbiome changes reduce hepatic copper directly or act mainly through immune activation, barrier repair, or both (18, 19). A similar issue applies to barrier biology. Although barrier dysfunction is a plausible amplifier, many studies rely on surrogate markers or static measurements rather than dynamic readouts of permeability linked to portal endotoxin flux and Kupffer cell activation, which are the kinds of data needed to establish mechanism more convincingly (62, 63, 100).

Renal involvement remains one of the largest gaps in a complete axis model. Clinical observations suggest that kidney injury in WD can be mechanistically primary, driven by direct copper toxicity rather than simply by advanced liver failure. Even so, the field still lacks a WD-specific causal framework connecting defined copper species exposure states to tubular stress programs and to systemic feedback through uremic solute accumulation and redox–inflammatory amplification (103, 109–113, 115). Cuproptosis is an appealing concept for mitochondrial-centered copper injury, but it still requires direct validation in renal tubules within WD models and careful integration with better established metalotoxic and inflammatory death pathways before it can be used with confidence in this setting (140).

At the brain interface, the main gap is not whether neuroinjury occurs, but how to connect systemic copper flux to specific neurologic phenotypes over time. Neuroimaging captures downstream structural damage reasonably well, yet longitudinal studies are still needed to relate those changes to upstream flux dynamics and interface states, and to distinguish persistent deposition effects from continuing immune–metabolic amplification. The blood–brain barrier (BBB) remains a plausible bottleneck on mechanistic grounds and is supported by *in vitro* data, but clinical biomarker panels that jointly track BBB integrity, copper speciation, and neurologic trajectories have not yet been established (117, 119, 122).

Methodologically, the field now needs study designs that can test mediation and directionality directly. A genuinely testable axis model requires concurrent measurement of exposure states, intermediate biology, and organ phenotypes within the same individuals over time. In practical terms, that means tracking bioavailable copper species and kinetic flux; microbiome composition, metabolites, gut permeability, endotoxin translocation, innate immune activation, and bile acid signaling; and downstream phenotypes such as fibrosis, renal tubular dysfunction, and neuroimaging changes in parallel. Only integrated longitudinal designs of this kind will move WD research beyond stacked correlations and toward causal mechanism (93, 118, 127).

## 9 Conclusions

WD is more usefully understood as a dynamic flux disorder than as a static disease of copper storage. Bioavailable copper species and systemic spillover initiate organ stress, and inter-organ feedback loops then sustain and amplify that injury. The gut–liver interface is the clearest example: copper-driven dysbiosis and epithelial barrier failure increase portal delivery of microbial products, which activates hepatic innate immunity, while altered bile acid signaling feeds back to maintain gut dysfunction even when systemic copper indices improve. The liver–kidney and liver–brain interfaces extend the same logic. Tubular vulnerability, BBB disruption, and metal-triggered inflammatory cell-death programs may maintain multi-organ risk despite partial biochemical control, offering a more convincing explanation for phenotypic heterogeneity than the traditional “copper-only” model.

This view also changes therapeutic priorities. Copper lowering remains fundamental, but durable disease modification will probably require interruption of loop gain at several nodes: restoring physiologic copper excretion, stabilizing the gut ecosystem and epithelial barrier, suppressing hepatic innate immune amplification, and protecting neurovascular interfaces, all with support from biomarker-anchored and imaging-based endpoints. Mechanistically defined multi-component pharmacological adjuncts may contribute meaningfully when they are mapped to specific axis nodes and assessed with modern biomarkers and imaging tools, as illustrated by a recent randomized, double-blind, placebo-controlled trial linking microbiome modulation to improvement of liver fibrosis indices in WD (101). In that sense, the gut–liver–kidney–brain axis shifts WD from a disease managed mainly by a single biochemical surrogate to a systems disorder defined by measurable fluxes and modifiable feedback loops, creating a clearer path toward biomarker-guided, node-specific, and trial-ready interventions.
