# Supplementary material for: The gut-liver-kidney-brain axis in Wilson disease: copper speciation-flux and barrier-mediated organ crosstalk
Source: Front Immunol. 2026 Jun 17;17:1840716. doi: 10.3389/fimmu.2026.1840716 (PMC13318724; doi:10.3389/fimmu.2026.1840716)
Supplement: Supplementary file 1 [file Table1.docx]

**The Gut-Liver-Kidney-Brain Axis in Wilson Disease: Copper Speciation-Flux and Barrier-Mediated Organ Crosstalk**

Table 1. Evidence Matrix for the Gut–Liver–Kidney–Brain Axis in Wilson Disease (WD)

| **Axis Interface** | **Mechanism/Link** | **Evidence Tier** | **Key Findings** | **Key References** |
| --- | --- | --- | --- | --- |
| Gut → Liver | Dysbiosis contributes causally to hepatic copper burden and injury | Animal Interventional + Human Cohort | In WD patients and Atp7b-deficient mice, Lactobacillus depletion associates inversely with liver injury; FMT from healthy donors restores Lactobacillus, reduces hepatic copper, and alleviates liver injury, whereas WD microbiota transfer aggravates injury, supporting causality rather than association | (1) |
| Gut → Liver | Multi-omics dysbiosis signature and metabolite depletion | Human Cohort | Multi-omics profiling demonstrates reduced microbial diversity with Megamonas/Selenomonaceae enrichment and Roseburia inulinivorans depletion; fecal metabolites with putative protective roles (e.g., leucylproline, 5-phenylvaleric acid) decrease, while D-gluconic acid correlates with specific taxa | (2) |
| Gut → Liver | Copper spillover reshapes microbiota and is reversible with intervention | Animal Interventional | In copper-overloaded WD models, Corynebacterium increases and Lactobacillus decreases; intervention restores Ligilactobacillus abundance in parallel with improved hepatic injury, reinforcing copper-driven dysbiosis as an actionable node | (3) |
| Gut → Liver | Barrier failure (“leaky gut”) from copper toxicity via tight junction loss and enterocyte mitochondrial injury | Human + Animal (Mechanistic/Observational) | Duodenal histology in WD shows villus blunting and lymphocytic infiltration; models show loss of tight junction/adhesion proteins (e.g., claudins/cadherins) and distinct enterocyte mitochondrial swelling/cristae rarefaction; methanobactin rescues barrier integrity and mitochondrial architecture | (4) |
| Gut → Liver | Microbial product translocation amplifies hepatic innate immune activation (Gut–LPS–Liver loop) | Mechanistic (Supported by barrier + dysbiosis evidence) | Convergent evidence that dysbiosis and barrier disruption create a permissive state for endotoxin/LPS translocation into portal circulation and promote hepatic inflammatory programs; causality is strengthened when microbiota correction ameliorates hepatic injury and copper burden | (1, 4) |
| Liver → Gut | Bile-acid/FXR axis reprogramming feeds back to shape gut ecology and inflammation | Animal Interventional + Human Validation | Copper overload contracts the hepatic FXR cistrome, downregulating canonical targets (e.g., BSEP, SHP) and producing bile-acid accumulation/composition shifts; parallel serum bile-acid changes in WD patients correlate with hepatic severity, supporting a liver→gut feedback conduit | (5) |
| Liver → Gut | Copper disrupts nuclear receptor signaling broadly (FXR/LXR/PPARα/HNF4α) with downstream metabolic and bile-acid transport consequences | Animal Mechanistic (Proteomics) | Quantitative proteomics in Atp7b−/− liver identifies early, global suppression of nuclear receptor target programs (RXR/LXR/FXR/PPARα/HNF4α), consistent with copper acting as a transcriptional disruptor that predisposes to cholestasis/steatosis and likely second-order gut feedback via bile-acid transport/signaling | (6) |
| Systemic (Gut→Liver as flux) | Copper flux and “systemic spillover” visualized in vivo | Human Imaging | ^64^Cu-PET/CT demonstrates rapid hepatic uptake (≈44% within the scanning window) and substantial extrahepatic distribution (≈45–58%), with renal uptake (2–6%) indicating filtration/reuptake of circulating non-ceruloplasmin-bound copper and providing a kinetic basis for multi-organ exposure | (7) |
| Systemic (Gut→Liver) | Real-time visualization of intestinal uptake and hepatic accumulation; zinc closes the intestinal “gate” | Imaging/Methodological (Human/Translational) | ^64^Cu-PET/CT tracer workflows visualize copper absorption and rapid liver sequestration; zinc therapy reduces hepatic tracer signal, giving direct visual support for intestinal uptake blockade as a gut-node therapy | (8) |
| Liver → Kidney | Clinical spectrum: tubular dysfunction, hypercalciuria/nephrocalcinosis as early presentation | Human Cohort/Case Evidence | WD can present with hypercalciuria and nephrocalcinosis before overt hepatic manifestations, consistent with proximal tubular vulnerability; renal tubular injury may secondarily perturb mineral handling and acidification | (9) |
| Liver → Kidney | Distal RTA–hypercalciuria–stone/hematuria chain reaction | Human Case Evidence | WD-associated distal RTA can alkalinize urine and promote calcium precipitation, linking copper toxicity to a recognizable renal complication cascade (RTA → hypercalciuria → stones/hematuria) | (10) |
| Liver ↔ Kidney | Renal copper handling is not passive “spillover”; tubular ATPase biology can drive luminal copper leakage | Animal Mechanistic | Kidney co-expresses ATP7A/ATP7B; in Atp7b−/− kidney, altered ATP7A trafficking/expression supports adaptive changes in basolateral reabsorption and suggests tubular sequestration failure and luminal leakage contributes to hypercupriuria beyond plasma spillover alone | (11) |
| Kidney (WD) | Renal interstitial fibrosis mechanism implicating leptin–JAK2–STAT3 signaling and therapeutic reversibility | Animal Interventional (Mechanistic) | In TX mice, renal involvement includes interstitial fibrosis with activation of the Leptin–JAK2–STAT3 pathway; intervention that suppresses JAK/STAT attenuates fibrosis, positioning renal fibrosis as a modifiable axis component | (12) |
| Gut ↔ Kidney ↔ Brain | Gut–brain–kidney axis via microbial metabolites/uremic toxins (analogous evidence; mechanistic scaffold for WD) | Review/Consensus (Analogous to CKD models) | A unified framework proposes dysbiosis-driven production of uremic toxins (e.g., indoxyl sulfate, p-cresyl sulfate) that stress renal tubular transport and can impact BBB/neuroinflammation; although developed for CKD, the “circulating metabolite toxicity” model offers a mechanistic bridge for concurrent neuro-renal vulnerability in WD when dysbiosis is present | (13) |
| Liver → Brain | Subclinical neurodegeneration in “hepatic phenotype” challenges the liver-only classification | Human Cohort (Imaging) | Brain morphometry in “hepatic WD” (no overt neuropsychiatric symptoms) shows reduced volumes in thalamus, globus pallidus, hippocampus, and brainstem, supporting early anatomical impact consistent with systemic spillover and thresholded symptom emergence | (14) |
| Blood → Brain (Copper species) | Copper–albumin complexes disrupt BBB tight junctions; bis-choline TTM neutralizes toxicity | In Vitro | Copper bound to albumin (Cu–Alb) compromises BBB tight junction proteins (e.g., ZO-1, occludin); bis-choline tetrathiomolybdate sequesters copper into inert complexes and prevents endothelial cytotoxicity, offering a BBB-protective rationale distinct from mobilizing chelators | (15) |
| Therapy → Brain risk | Chelation initiation can transiently increase free copper and oxidative stress in brain (“paradoxical worsening”) | Animal Mechanistic | In toxic milk mice, penicillamine induces an early surge in circulating free copper, increased brain copper and lipid peroxidation (MDA), providing a mechanistic basis for early neurologic worsening during copper mobilization | (16) |
| Brain phenotypes | Predominant brainstem involvement can occur and drives diagnostic pitfalls | Human Case Evidence | WD can present with symmetric brainstem T2/FLAIR hyperintensities with relative basal ganglia sparing, mandating careful differentiation from demyelination/encephalitis and reinforcing reliance on copper metabolic evaluation | (17) |
| Brain imaging (advanced) | High-field susceptibility imaging distinguishes mixed metal deposition patterns | Human Imaging | 7T SWI identifies a distinctive putaminal pattern (hypointense core with hyperintense rim) consistent with mixed paramagnetic/diamagnetic contributors, aiding differentiation from NBIA and refining the “metal deposition phenotype” | (18) |
| Brain imaging (advanced) | QSM in WD models can separate copper- vs iron-like signatures | Animal Imaging | 9.4T MRI-QSM in TX mice shows copper accumulation regions with decreased (negative) susceptibility values, supporting a tractable imaging strategy for in vivo copper mapping | (19) |
| Brain activity vs chronic damage | MRI scales separate acute toxicity (reversible) from chronic damage (irreversible) and correlate with UWDRS | Human Cohort (Imaging) | Semiquantitative MRI scales partition acute toxicity (T2 hyperintensity/edema; treatment-responsive) versus chronic damage (atrophy/T2 hypointensity; less reversible) with strong correlations to neurologic severity scores | (20) |
| Neuro-heterogeneity & misdiagnosis | WD as the “great imitator” and biomarker pitfalls during inflammation | Review/Consensus (Clinical) | Neurologic WD can mimic parkinsonism/dystonia/psychiatric disease; ceruloplasmin may appear falsely normal during acute inflammation, underscoring diagnostic vulnerability and motivating multi-marker approaches | (21) |
| TCM/Integrative | Dysphagia improvement with antioxidant shifts (MDA↓, SOD↑) in RCT context | Human Interventional | In WD with dysphagia, integrative therapy plus training lowers serum MDA, increases SOD, and improves swallowing function scores, consistent with oxidative stress modulation as a brain-node supportive mechanism | (22) |
| Cell death (analogous) | Cuproptosis machinery as a transferable concept for copper-driven injury (not WD-specific) | Hypothetical/Analogous | Cuproptosis correlates with macrophage/foam-cell death and plaque instability in atherosclerosis; although not WD-specific, the pathway highlights how bioavailable copper can trigger regulated death programs that may generalize to copper-exposed tissues | (23) |

Table 2. Copper Species, Measurement Approaches, and Diagnostic/Translational Utility

| **Copper Species** | **Physiological Meaning** | **Diagnostic/Monitoring Utility** | **Limitations** | **Key References** |
| --- | --- | --- | --- | --- |
| Total serum copper | Aggregate circulating copper (ceruloplasmin-bound + non-ceruloplasmin fractions) | Broad screening adjunct; can support acute fulminant phenotypes when interpreted with other markers | Can be misleading in WD because total copper may fall when ceruloplasmin is low; does not isolate the toxic/bioavailable fraction | (8) |
| Ceruloplasmin (immunoreactive) | Protein quantity measured by immunoassay; may include apo-ceruloplasmin (functionally inactive) | Traditional diagnostic marker; widely available | Immunoreactive ceruloplasmin can be falsely normal (e.g., during inflammation) and can overestimate functional copper-binding capacity because apo-CP is detected | (21, 24) |
| Ceruloplasmin oxidase activity (functional CP) | Enzymatic activity reflecting functional ceruloplasmin (holoprotein performance) | Improves diagnostic accuracy compared with immunoreactive CP; high AUC reported and reduces false negatives in functionally deficient states | Requires enzymatic assay infrastructure; cutoffs and standardization needed across labs | (24) |
| NCC (non-ceruloplasmin-bound copper; “free”/bioavailable copper pool, operational definitions vary) | Copper not carried by ceruloplasmin, often modeled as a readily exchangeable/toxic fraction | Conceptual measure of systemic toxicity and spillover; connects mechanistically to extrahepatic exposure and BBB injury paradigms | Calculated NCC depends on assay accuracy and assumptions; “free copper” can be method-dependent and unstable; terminology inconsistencies across studies | (7, 8, 15) |
| Exchangeable copper / REC (Relative Exchangeable Copper) | Fraction of circulating copper that is bioavailable/exchangeable relative to total; intended to represent toxic spillover more faithfully than calculated “free copper” | Proposed as a robust toxicity metric; REC > 18.5% highlighted as a superior indicator of systemic copper burden/spillover and a candidate for monitoring response and risk stratification | Requires specialized methodology; thresholds need harmonization; adoption not yet universal | (8) |
| ^64^Cu-PET/CT flux imaging (functional copper kinetics) | Real-time visualization of copper distribution/uptake as a kinetic “flux phenotype” rather than a static concentration | Quantifies systemic spillover (extrahepatic distribution), identifies kidney uptake consistent with NCC filtration/reuptake, and provides a translational readout of gut-node therapies (e.g., zinc reducing hepatic tracer signal) | Limited availability, radiation exposure, tracer logistics; primarily research/advanced-center tool today | (7, 25) |

Table 3. Mapping Evidence and Translational Pathways of the Gut–Liver–Kidney–Brain Axis in Wilson Disease

| **Axis Node** | **Human Clinical Evidence** | **Causal/Interventional Evidence** | **Molecular Mechanism** | **Translational Biomarkers** | **Evidence Strength Rating** |
| --- | --- | --- | --- | --- | --- |
| **Gut Node & Interface (Dysbiosis, Barrier, Portal Influx)** | WD multi-omics defines a reproducible dysbiosis–metabolite signature with reduced diversity, enrichment of Selenomonaceae/Megamonas, depletion of the butyrate producer Roseburia inulinivorans, and concordant shifts in fecal metabolites including decreased leucylproline and 5-phenylvaleric acid, with taxon–metabolite correlations (e.g., D-gluconic acid) (2). Cross-species concordance shows reduced Lactobacillus in WD patients and WD models, linking dysbiosis to liver injury severity in humans (1). Human duodenal pathology supports a true barrier phenotype with villus blunting and lymphocytic infiltration (4). | Healthy-donor FMT causally rescues the gut–liver phenotype in Atp7b deficiency by restoring Lactobacillus, reducing hepatic copper burden, and alleviating liver injury, whereas WD-microbiota transfer worsens injury, directly supporting directionality (microbiome → liver copper/injury) (1). Copper-directed therapy (methanobactin) reverses intestinal barrier damage and enterocyte mitochondrial pathology, supporting copper causality for barrier failure and therapeutic reversibility at the gut interface (4). | Copper overload disrupts epithelial junctional architecture (tight junction/adherens junction protein loss; claudin/cadherin family changes) and induces node-specific enterocyte mitochondrial swelling/cristae disruption that plausibly drives permeability “leak” (4). Copper can directly suppress AQP3 permeability in intestinal epithelial context, providing a concrete molecular handle for epithelial dysfunction under copper stress (26). Copper-driven dysbiosis has a newly supported signature in copper-overload rats with Corynebacterium enrichment and Lactobacillus depletion, with intervention restoring Ligilactobacillus, consistent with copper-conditioned ecological selection pressures (3). A portal-influx amplification scaffold is supported by WD endotoxemia biology showing altered hepatic immune responses to LPS-like challenges, mechanistically consistent with gut-derived PAMP delivery as a loop amplifier even when direct portal LPS quantification is not performed in the same studies (27). | Microbiome composition (Roseburia depletion; Lactobacillus depletion; Corynebacterium enrichment) as stratifiers (1-3). Fecal metabolite panels including leucylproline and 5-phenylvaleric acid as functional readouts of ecological remodeling (2). Duodenal histology and junctional integrity proteins as barrier readouts (4). | Strong/Causal for dysbiosis→liver injury via FMT and for barrier reversibility under copper-directed therapy (1, 4); Emerging for specific portal PAMP quantification within WD cohorts despite strong mechanistic plausibility from endotoxemia paradigms (27). |
| **Liver Node (Inflammation, Metabolism, Bile Feedback)** | In clinically stable/treated WD, persistent macrophage/Kupffer activation is detectable, with elevated sCD163 correlating inversely with metabolic liver function (galactose elimination capacity) rather than stiffness-defined fibrosis stage, indicating an immune-activation state that can persist beyond classic “copper control” (28). Serum bile-acid profiles in WD patients associate with liver disease severity, and total bile acids predict severe hepatic disease, supporting bile acids as a clinically measurable feedback axis (5, 29). | Nuclear receptor modulation provides interventional proof that copper load alone does not fully define injury severity: LXR agonism reduces hepatic inflammation and fibrosis and restores metabolic balance in Atp7b−/− mice despite persistently high hepatic copper, demonstrating that inflammatory–metabolic programs can be therapeutically uncoupled from copper concentration (30). Hepatocyte-only ATP7B loss yields marked copper accumulation but only mild/delayed inflammation, implying that non-parenchymal/interface signals (consistent with gut-derived or systemic cues) are required to ignite full inflammatory escalation (31). | Copper contracts the FXR cistrome, suppresses canonical FXR targets (e.g., Bsep, Shp), and drives bile-acid accumulation and compositional shifts, providing a direct molecular bridge to liver→gut feedback and to cholestatic/metabolic remodeling (5). Systems proteomics demonstrates early global suppression of nuclear receptor networks (RXR/LXR/FXR/PPARα/HNF4α) in Atp7b−/− liver, supporting copper as a transcriptional disruptor with downstream metabolic consequences that can feed back to the gut through bile-acid signaling and transport alterations (6). | sCD163 as an immune activation/Kupffer readout that tracks liver functional capacity (28). Serum bile acids (including total bile acids) as severity-linked metrics reflecting FXR/bile feedback dysfunction (5, 29). | Emerging/Correlative for human immune activation and bile acids as axis readouts (strong association but limited causal human perturbation) (5, 28, 29); Strong/Causal for mechanistic FXR disruption and interventional nuclear-receptor modulation in WD models (5, 6, 30). |
| **Kidney Node (Tubulopathy, Systemic Feedback)** | WD renal involvement spans clinically meaningful tubulopathy phenotypes, including early hypercalciuria/nephrocalcinosis that can precede overt hepatic recognition (9) and distal RTA-associated urine alkalinization with downstream stone/hematuria complications (10). Phenotyping must consider diagnostic confounding: proteinuria can artifactually lower ceruloplasmin and elevate urinary copper, risking misclassification without genetic or hepatic copper confirmation (32). | In TX mice, renal interstitial fibrosis is mechanistically linked to the leptin–JAK2–STAT3 axis and is attenuated by intervention targeting this pathway, providing causal evidence that renal remodeling is not purely secondary but can be pathway-driven and modifiable in WD contexts (12). | Kidney copper handling is mechanistically active: ATP7A/ATP7B expression and ATP7A trafficking changes under ATP7B deficiency support altered basolateral reabsorption and luminal copper leakage, offering a mechanistic substrate for hypercupriuria and tubular stress beyond passive systemic spillover (11). Copper can inhibit AQP3 permeability at micromolar concentrations, providing a plausible epithelial transport mechanism relevant to distal nephron vulnerability and acid–base/solute handling disturbances (26). A systems amplification scaffold (gut–kidney–brain metabolite toxicity) is defined in the uremic-toxin framework of the gut–brain–kidney axis in CKD, which is mechanistically analogous and proposes microbial metabolite traffic as a mediator of systemic and neurovascular injury; this remains extrapolative in WD unless directly validated (13). | Renal phenotype readouts: hypercalciuria/nephrocalcinosis imaging and urine chemistries (phenotype anchors) (9, 10). Fibrosis pathway activity markers centered on JAK/STAT signaling in experimental contexts (12). | Emerging for human renal spectrum linkage to systemic axis outcomes (strong clinical observations but limited WD-native mediation studies) (9, 10, 32); Strong/Causal for renal fibrosis mechanism in WD model (JAK2/STAT3) and for defined copper-transport remodeling biology (11, 12); Hypothetical for uremic-toxin feedback to brain in WD (imported/analogous CKD axis) (13). |
| **Brain Node (BBB, Neuroinflammation, Direct Toxicity)** | WD neurologic heterogeneity creates recurrent diagnostic pitfalls (“great imitator”), with potential false reassurance from ceruloplasmin appearing normal during inflammation and with presentations mimicking parkinsonism/dystonia/psychiatric disease (21). Subclinical brain involvement can occur even in “hepatic WD,” with morphometry showing reduced volumes in thalamus, globus pallidus, hippocampus and brainstem despite absent overt neuropsychiatric symptoms, supporting early axis penetration of the brain node (14). | Therapy-phase flux dynamics can worsen neurotoxicity: penicillamine initiation in WD models increases circulating free copper, raises brain copper, and induces oxidative stress signatures, supporting a causal “flux overshoot” mechanism for paradoxical neurologic worsening (16). BBB-targeted copper sequestration is supported by experimental BBB models showing that copper–albumin injures tight junction architecture and that bis-choline tetrathiomolybdate neutralizes toxicity, implying a causal route for reducing exposure at the barrier (15). | Copper–albumin disrupts BBB tight junction components (e.g., ZO-1/occludin architecture) and induces endothelial cytotoxicity that can be prevented by copper sequestration into inert complexes (bis-choline tetrathiomolybdate), providing a defined barrier mechanism (15). Neuroinflammation and oxidative stress represent actionable downstream programs: melatonin activates SIRT3/FOXO3a, normalizes autophagy tone, suppresses NLRP3/IL-1β signaling, and improves cognitive phenotypes in WD contexts (33). Integrative neuro-rehabilitation evidence supports modifiable redox biology, with reduced MDA, increased SOD, and improved dysphagia scores in a controlled clinical study (22). | Neuro-heterogeneity framing and diagnostic vigilance (clinical + laboratory integration) (21). Brain structural endpoints (morphometry volumes) as early phenotype readouts (14). Oxidative stress markers (MDA, SOD) coupled to functional endpoints in interventional context (22). BBB integrity as a mechanistic endpoint inferred from tight junction preservation under copper sequestration (15). | Emerging for human heterogeneity and occult brain involvement (strong clinical/imaging associations but limited mechanistic mediation in patients) (14, 21); Strong/Causal for therapy-phase flux overshoot and BBB copper-toxicity mechanism with pharmacologic rescue in experimental systems (15, 16); Emerging for oxidative stress modulation translating to clinical functional improvement (22). |

1. Zhong HJ, Liu AQ, Huang DN, Zhou ZH, Xu SP, Wu L, Yang XP, et al. Exploring the impact of gut microbiota on liver health in mice and patients with Wilson disease. Liver Int 2024;44:2700-2713.

2. Cai X, Dai J, Xie Y, Xu S, Liu M. Multi-omics study unravels gut microbiota and metabolites alteration in patients with Wilson's disease. Sci Rep 2024;14:21025.

3. Zhuang T, Meixia W, Gang W, Jiafeng Z, Shuai K, Rui W, Wenming Y. Characteristics of gut microbiota in copper-loaded rats with Wilson's disease and the intervention effect of Hepatol and Fumu Decoction. China Journal of Traditional Chinese Medicine and Pharmacy 2025;40:841-847.

4. Fontes A, Pierson H, Bierla JB, Eberhagen C, Kinschel J, Akdogan B, Rieder T, et al. Copper impairs the intestinal barrier integrity in Wilson disease. Metabolism 2024;158:155973.

5. Wooton-Kee CR, Yalamanchili HK, Mohamed I, Hassan M, Setchell KDR, Narvaez Rivas M, Coskun AK, et al. Changes in the FXR-cistrome and alterations in bile acid physiology in Wilson disease. Hepatol Commun 2025;9.

6. Wilmarth PA, Short KK, Fiehn O, Lutsenko S, David LL, Burkhead JL. A systems approach implicates nuclear receptor targeting in the Atp7b(-/-) mouse model of Wilson's disease. Metallomics 2012;4:660-668.

7. Munk DE, Vendelbo MH, Kirk FT, Rewitz KS, Bender DA, Vase KH, Munk OL, et al. Distribution of non-ceruloplasmin-bound copper after i.v. (64)Cu injection studied with PET/CT in patients with Wilson disease. JHEP Rep 2023;5:100916.

8. Roberts EA, Schilsky ML. Current and Emerging Issues in Wilson's Disease. N Engl J Med 2023;389:922-938.

9. Di Stefano V, Lionetti E, Rotolo N, La Rosa M, Leonardi S. Hypercalciuria and nephrocalcinosis as early feature of Wilson disease onset: description of a pediatric case and literature review. Hepat Mon 2012;12:e6233.

10. Sinha R, Akhtar S. Gross hematuria in a case of Wilson disease: answers. Pediatr Nephrol 2012;27:919-920; 917.

11. Linz R, Barnes NL, Zimnicka AM, Kaplan JH, Eipper B, Lutsenko S. Intracellular targeting of copper-transporting ATPase ATP7A in a normal and Atp7b-/- kidney. Am J Physiol Renal Physiol 2008;294:F53-61.

12. Xiang L, Wenming Y, Yue Y, Wenjie H, Peipei L, Jianpeng H. The intervention effect of Gan Dou Fu Mu Tang on renal fibrosis in TX mice through the JAK/STAT signaling pathway. Chinese Journal of Experimental Traditional Medical Formulae 2023;29:26-35.

13. Zhu J, Fu Y, Olovo CV, Xu J, Wu Q, Wei W, Jiang K, et al. The influence of gut microbiota on the gut-brain-kidney axis and its implications for chronic kidney disease. Front Microbiol 2025;16:1535356.

14. Rahimi P, Mareček S, Brůha R, Dezortová M, Sojka P, Hájek M, Skowrońska M, et al. Brain morphometry in hepatic Wilson disease patients. J Inherit Metab Dis 2025;48:e12814.

15. Borchard S, Raschke S, Zak KM, Eberhagen C, Einer C, Weber E, Müller SM, et al. Bis-choline tetrathiomolybdate prevents copper-induced blood-brain barrier damage. Life Sci Alliance 2022;5.

16. Chen DB, Feng L, Lin XP, Zhang W, Li FR, Liang XL, Li XH. Penicillamine increases free copper and enhances oxidative stress in the brain of toxic milk mice. PLoS One 2012;7:e37709.

17. Zhou L, Zhao C, Man X, Sun Z, Xiang Y. Brainstem Involvement in a Patient with Wilson Disease. Neurol India 2024;72:686.

18. Su D, Zhang Z, Zhang Z, Zheng S, Yao T, Dong Y, Zhu W, et al. Distinctive Pattern of Metal Deposition in Neurologic Wilson Disease: Insights From 7T Susceptibility-Weighted Imaging. Neurology 2024;102:e209478.

19. Han Y, Dong J, Xu C, Rao R, Shu S, Li G, Cheng N, et al. Application of 9.4T MRI in Wilson Disease Model TX Mice With Quantitative Susceptibility Mapping to Assess Copper Distribution. Front Behav Neurosci 2020;14:59.

20. Dusek P, Smolinski L, Redzia-Ogrodnik B, Golebiowski M, Skowronska M, Poujois A, Laurencin C, et al. Semiquantitative Scale for Assessing Brain MRI Abnormalities in Wilson Disease: A Validation Study. Mov Disord 2020;35:994-1001.

21. Roy D, Mukherjee A, Chakravarty A. Pitfalls in the Diagnosis of Wilson Disease. Curr Neurol Neurosci Rep 2025;25:40.

22. Shiqiao, Meixia W, Nannan Q, Ningshu S, Mingzhu N, Wenming Y. Observation on the efficacy of liver bean and bupleurum decoction combined with swallowing training for dysphagia in Wilson's disease. Journal of Nanjing University of Traditional Chinese Medicine 2025;41:115-121.

23. Muhetaer M, He T, Zhu H, Wu J, Wan J, Zhang T, Hu Y, et al. Analysis of the Correlation Between Cuproptosis and Instability of Atherosclerotic Plaques. Biomedicines 2025;13.

24. Yang Y, Cheng T, Yang W, Wang Y, Yang Y, Xi H, Zhu Q. Serum ceruloplasmin oxidase activity: A neglected diagnostic biomarker for Wilson disease. Parkinsonism Relat Disord 2024;127:107105.

25. Emilie Munk D, Teicher Kirk F, Vendelbo M, Vase K, Munk O, Ott P, Damgaard Sandahl T. Positron Emission Tomography Using 64-Copper as a Tracer for the Study of Copper-Related Disorders. J Vis Exp 2023.

26. Zelenina M, Tritto S, Bondar AA, Zelenin S, Aperia A. Copper inhibits the water and glycerol permeability of aquaporin-3. J Biol Chem 2004;279:51939-51943.

27. Cichon I, Ortmann W, Bednarz A, Lenartowicz M, Kolaczkowska E. Reduced Neutrophil Extracellular Trap (NET) Formation During Systemic Inflammation in Mice With Menkes Disease and Wilson Disease: Copper Requirement for NET Release. Front Immunol 2019;10:3021.

28. Björklund J, Laursen TL, Sandahl TD, Møller HJ, Vilstrup H, Ott P, Grønbæk H. High hepatic macrophage activation and low liver function in stable Wilson patients - a Danish cross-sectional study. Orphanet J Rare Dis 2018;13:169.

29. Xiao QQ, Xu YH, Xu X, Shi YW, Cao HX, Liu XQ, Fan JG. [Analysis of clinical and genetic characteristics of the severe liver disease phenotype in patients with hepatolenticular degeneration]. Zhonghua Gan Zang Bing Za Zhi 2024;32:551-557.

30. Hamilton JP, Koganti L, Muchenditsi A, Pendyala VS, Huso D, Hankin J, Murphy RC, et al. Activation of liver X receptor/retinoid X receptor pathway ameliorates liver disease in Atp7B(-/-) (Wilson disease) mice. Hepatology 2016;63:1828-1841.

31. Muchenditsi A, Yang H, Hamilton JP, Koganti L, Housseau F, Aronov L, Fan H, et al. Targeted inactivation of copper transporter Atp7b in hepatocytes causes liver steatosis and obesity in mice. Am J Physiol Gastrointest Liver Physiol 2017;313:G39-g49.

32. Khan S, Schilsky M, Silber G, Morgenstern B, Miloh T. The Challenges of Diagnosing and Following Wilson Disease in the Presence of Proteinuria. Pediatr Gastroenterol Hepatol Nutr 2016;19:139-142.

33. Wang L, Wu L, Wang T, Yue Y, Jiang Z, Jiang P, Zhou H, et al. Melatonin ameliorates copper accumulation-induced cognitive impairment in Wilson disease via activation of the SIRT3/FOXO3α signaling pathway. Neuropharmacology 2026;284:110779.
